# Constituent Characteristics and Functional Properties of Passion Fruit Seed Extract

**DOI:** 10.3390/life12010038

**Published:** 2021-12-27

**Authors:** Shinpei Kawakami, Makoto Morinaga, Sakuka Tsukamoto-Sen, Sadao Mori, Yuko Matsui, Toshihiro Kawama

**Affiliations:** Health Science Research Center, R & D Institute, Morinaga & Co., Ltd., 2-1-1 Shimosueyoshi, Tsurumi-ku, Yokohama 230-8504, Japan; m-morinaga-bj@morinaga.co.jp (M.M.); s-tsukamoto-ad@morinaga.co.jp (S.T.-S.); s-mori-ab@morinaga.co.jp (S.M.); y-matsui-jd@morinaga.co.jp (Y.M.); t-kawama-jb@morinaga.co.jp (T.K.)

**Keywords:** *Passiflora edulis*, seed, passion fruit, polyphenol, stilbenes, piceatannol, antioxidant

## Abstract

The genus *Passiflora* L. is widely cultivated in tropical and subtropical regions. The major species, *Passiflora edulis* Sims, is known as ‘passion fruit’ and is widely used in processed foods as well as eaten raw. *P. edulis* fruits are eaten for their pulp together with the seeds; however, the seeds are often discarded when used in processed foods. *P. edulis* seeds contain a variety of nutrients and functional components, and their industrial use is desirable from the perspective of waste reduction. Previous studies have analyzed the constituents of *P. edulis* and their physiological functions. *P. edulis* seeds contain various types of polyphenols, especially those rich in stilbenes (e.g., piceatannol). *P. edulis* seed extracts and isolated compounds from seeds have been reported to exhibit various physiological functions, such as antioxidant effects, improvement of skin condition, fat-burning promotion effects, and hypoglycemic effects. This review summarizes the nutritional characteristics, polyphenol content, and physiological functions of *P. edulis* seeds.

## 1. Introduction

The genus *Passiflora* L. is a highly diverse plant family with approximately 520 species distributed throughout the tropics of America, Asia, and Africa [1]. More than 90% of *Passiflora* species are distributed in the Americas; however, they are also widely distributed in India, China, Southeast Asia, Australia, the Pacific islands, and neighboring regions [2]. *Passiflora* fruits vary in color and shape and are mostly berries with a few pulpy capsules. The fruits of most species are edible; however, only five or six species are economically important [1]. *Passiflora edulis* Sims is commonly known as the passion fruit, and the most cultivated species globally include the yellow passion fruit (*P. edulis* f. *flavicarpa*) and purple passion fruit (*P. edulis* f. *edulis*) [3]. Indeed, these two passion fruit species have been analyzed using metabolomics and transcriptomics to clarify the underlying mechanisms of color tone formation [4]. The concentrations of most of the flavonols, anthocyanins, and flavanols involved in color formation of fruit were significantly higher in purple passion fruits than in yellow passion fruits, and the characteristics of each passion fruit were clarified by molecular biological analysis.

Global passion fruit production was estimated as 1.5 million tons in 2017 [5]. Brazil is the largest producer, reaching 690.4 thousand tons in 2020 [6]. Passion fruit is a sour fruit that is split open, and the pulp inside is eaten raw with the seeds. In Brazil, which is one of the main producers and consumers of passion fruit, this fruit is widely used not only for eating raw, but also for making concentrates and juices [7]. Passion fruit is an attractive and nutritious fruit that is highly appreciated for its diverse uses, such as juices, jellies, and ice cream products, for fresh consumption, and in industrial use [8]. Furthermore, passion fruit is used in healthcare products and pharmaceuticals, and passion fruit industry is expected to develop in the future owing to its popularity and growing production [9]. When processing passion fruit into juice and other products, peels and seeds are produced as by-products; therefore, the larger the production scale, the larger the amount of by-products [10]. Since these residues represent an operating cost to industry and can be a significant burden to the environment, industrial use of the by-products is desirable.

*Passiflora* species have long been thought to have anxiolytic and antidepressant properties and have been used as herbal medicines since ancient times [11,12]. The *Passiflora* plant can be divided into pulp, peel, seeds, and bark, the constituents and health benefits of each have been investigated, particularly for *P. edulis*. The extract of the edible portion reportedly has protective effects against alcoholic liver disease [13]. Moreover, leaf extract has shown a variety of physiological functions, such as being anti-inflammatory [14]; providing intestine protection [15]; and having wound healing [16], antiplatelet [17], and antidepressant effects [18]. It has also been evaluated in animal studies for its safety when administered [19]. *P. edulis* peel is rich in dietary fiber and functional components, and various physiological effects of *P. edulis* peel extract have been reported, such as antihypotensive effects [20], hypoglycemic effects [21,22], and metabolic improvement [23,24]. Furthermore, *P. edulis* bark reportedly has anti-obesity properties [25].

In this review, we focus on *P. edulis* seeds. Seeds store nutrients for embryo growth and are rich in fats, starches, proteins, and minerals. *P. edulis* seeds are edible and have interesting nutritional and biochemical properties that have nutritional and health benefits [26]; however, the seeds produced during processed food production are generally discarded after crushing [27]. For instance, in Brazil, >40% of passion fruit production is allocated to the juice and pulp industry, which produces large quantities of bagasse, including seeds [3]. Meanwhile, the seed cake, which is discarded after cold pressing seeds, contains fatty acids and phenolic compounds of interest [28]. *P. edulis* seeds are considered to be a valuable nutritional and functional material, and various studies on their functionality have been conducted. In this review, we summarize recent studies reporting the nutritional characteristics and functional components of *P. edulis* seeds and their functions. In particular, we summarize the characteristics and functions of stilbene polyphenols, which are characteristic of the seeds’ functional components.

## 2. *Passiflora edulis* Seed Compounds

### 2.1. Nutritional Composition of P. edulis Seeds

Previous studies have analyzed the nutrient and mineral composition of *P. edulis* seeds, and the results are summarized in Table 1.

Although there are differences among reports, *P. edulis* seeds contain a large amount of carbohydrates (49–71 g/100 g dry seeds), with >50% of the seeds composed of carbohydrates. Dry seeds also contain approximately 12–33 g lipids and 12–18 g protein per 100 g. The lipid content in *P. edulis* pulp and peel is 1–5 g/100 g (dry basis) and the protein content is approximately 6 g/100 g (dry basis) [30]; therefore, the seeds have higher percentages of lipids and protein than other plant parts. Pasflin, which exhibits antifungal effects, has been isolated and identified from *P. edulis* seeds [31]. Seeds also contain at least 10% protein and therefore can be used as a valuable protein source [26,29,30]. They are also rich in dietary fiber (48–66 g/100 g dry seeds) and are expected to be used as a source of dietary fiber. Some authors compared the constituents of yellow passion fruit (*P. edulis* f. *flavicarpa*) and purple passion fruit (*P. edulis* f. *edulis*) and reported no significant difference in the nutritional content of the seeds [30]. However, differences exist in the nutrient values presented in previous reports. Production systems (conventional and organic), plant nutritional status, production areas, and species heritability may affect the accumulation of vitamins and minerals in *P. edulis* seeds; however, these relationships are not well understood.

The lipid composition in *P. edulis* seeds is shown in Table 2. The lipid content in *P. edulis* was similar among the various studies. *P. edulis* seeds contain the highest amount of linoleic acid (i.e., a polyunsaturated fatty acid), accounting for approximately 70% of seed oil. A moderate intake of linoleic acid is associated with a lower risk of cardiovascular disease, most likely as a result of lower blood cholesterol concentrations [32]. The percentage of linoleic acid in the seeds of *P. pinnatistipula* Cav., *P. tripartita* (Juss.) Poir. and *P. ligularis* Juss., other species of *Passiflora*, is also reported to be about 70% [33]. Meanwhile, the percentage of linoleic acid in the seeds of *Cyphomandra betacea* Cav., one of the famous fruits in South America, is 58.3% [33], which is lower than that of *P.edulis*. *P. edulis* seeds also contain oleic acid, and the seed oil has a high unsaturated fatty acid content. In fact, the oil extracted from the seeds has been considered for commercial applications in the cosmetic, chemical, and pharmaceutical industries [10].

The mineral contents of *P. edulis* seeds are summarized in Table 1. However, the sodium content differs greatly among the reports and requires further analysis. Conversely, the content of minor minerals did not differ considerably among the reports. *P. edulis* seeds contain iron, copper, manganese, and zinc as minor minerals. These minerals are necessary elements for human physiological processes; for example, iron is a crucial component involved in tissue oxygenation and is a very important element, especially for pregnant women and infants [36]. The iron content in *P. edulis* seeds (4.30–7.27 mg/100 g seeds) is higher than that in maize, sunflower, or pumpkin seeds (4.2, 3.9, and 3.8 mg/100 g seeds, respectively) [26]; hence, *P. edulis* seeds can be useful as a source of iron supplementation.

There are few reports on vitamin analyses; oil extracted from *P. edulis* seeds using petroleum ether and diethyl ether has been analyzed, and α, β, γ, δ-tocopherol, and γ-tocotrienol were detected, with a high percentage of δ-tocopherol reported [10,37]. Moreover, analysis of the acetone-hexane extract detected 57.93 μg/100 g seeds of β-carotene, a vitamin A precursor [38].

### 2.2. Polyphenol Components in P. edulis Seeds

Analysis of total polyphenol contents in *P. edulis* peel, pulp, and seeds showed that 88% of the total polyphenols were found in the seeds [39]. Various polyphenols (including stilbenes) have been isolated and identified from seeds, and the reported polyphenol components are shown in Table 3.

There are many reports on the isolation of stilbenes from *P. edulis* seeds, especially piceatannol (3,3′,4,5′-tetrahydroxy-trans-stilbene), which is considered to be one of the main components of *P. edulis* seeds. Piceatannol was previously reported as a strong protein-tyrosine kinase inhibitor [50] and recently has become known as an activator of sirtuin (SIRT), which is one of the factors that regulate energy metabolism [51,52]. Piceatannol has been reported to be present in some plants, and the piceatannol content in the edible part of the grape berry is reported to be 0.78 µg/g [53]. According to studies on the amount of piceatannol contained in *P. edulis* seeds determined by ethanol extraction, concentrations of 4.8 [39], 5.7 [46], 13.97 [43], and 36.8 mg [29] per gram of dry seed were reported. Therefore, compared to other plants, the edible portion of *P. edulis* seeds is rich in piceatannol. The differences in the piceatannol content in *P. edulis* seeds reported among studies (4.8–36.8 mg piceatannol/g dry seeds) may be due to differences in extraction solvents and conditions, as well as the region of origin and harvest season of *P. edulis*. In addition to piceatannol, resveratrol (i.e., an SIRT activator) is also present in *P. edulis* seeds. Resveratrol is detected in ethanol and acetone extraction and is reported to be more efficiently extracted with acetone than ethanol [44]. In addition, the seeds also contain derivatives of piceatannol and resveratrol, such as scirpusin B, cassigarol D, cyperusphenol B, cyperusphenol D, astringin, piceid, pinostilbene, and gnetin C [43,45,46]. Scirpusin B, cassigarol D, cyperusphenol B, and cyperusphenol D are reported to have α-glucosidase inhibitory activities [43]. Scirpusin B, which is a dimer of piceatannol, has also been reported to exhibit strong vasorelaxant effects [46].

Several compounds classified as phenolic acids have also been found in *P. edulis* seeds. Caffeic acid, chlorogenic acid, ferulic acid, gallic acid, and rosmarinic acid have been detected in seeds when extracted with methanol [47], whereas coumarin and p-coumaric acid were detected in seeds when extracted with acetone and ethanol [38,45,48]. The coumarin and p-coumaric acid contents in seeds were reported to be 0.6 mg/g and 96 µg/g dry seeds, respectively [38,48], which is lower than the piceatannol content. In the seeds of *P. pinnatistipula*, *p*-coumaric acid was detected, while ferulic acid and gallic acid were not [33].

Various flavonoids have been detected in *P. edulis* seeds, mainly when extracted with ethanol. Aglycones such as epicatechin, quercetin, and kaempferol—as well as glycosides such as rutin, isoquercetin, malvidin 3,5-diglucoside, orientin, isoorientin, vitexin, and isovitexin—have been detected in *P. edulis* seeds [30,45,47,48,49]. As for the flavonoid content in seeds, kaempferol is relatively high at 3.75 mg/g seeds [30]. In comparison with the contents in other plants, the seeds of *Carthamus tinctorius* L. and *Phaseolus vulgaris* L. contain 0.8 mg/g and 13.8–209.4 µg/g of kaempferol, respectively [54,55]; thus, the seeds of *P. edulis* have a higher kaempferol content than the seeds of these species. Kaempferol has various physiological functions such as anti-cancer, antioxidant, anti-inflammatory, and neuroprotective properties [56]. Another flavonoid was reported to contain 421.56 and 341.59 μg/g seeds of isovitexin and vitexin, respectively [49]. Compounds such as isoorientin, isovitexin, and orientin have also been detected in *P. edulis* peels [57] and leaf extracts [18], suggesting that these compounds are widely localized in various parts of *P. edulis*.

## 3. Health Benefits of *P. edulis* Seed Components

### 3.1. Antioxidant Activity

*P. edulis* seeds contain a large amount of antioxidants such as polyphenols, and the seed extract has been reported to have high antioxidant activity in the 1,1-diphenyl-2-picrylhydrazyl (DPPH), 2,2′-azino-di-(3-ethylbenzthiazoline sulfonic acid) (ABTS), ferric reducing ability of plasma, oxygen radical absorbance capacity, and the β-carotene bleaching assays [29,47,58]. Santana et al. extracted components from *P. edulis* seeds under various extraction conditions and investigated the correlation between the component content and antioxidant activity under each condition [29]. The results showed a positive correlation between polyphenol content and antioxidant activity of the extracts, suggesting that polyphenols are a major component responsible for the antioxidant activity. Comparing the antioxidant activity of the seed extract of *P. edulis* with that of other *Passiflora* species, the IC50 of *P. edulis*, *P. tripartita*, *P. ligularis*, and *P. pinnatistipula* were 2.7–132.6, 3.2, 73.9, and 372.2, respectively, as determined via DPPH assay; meanwhile, the IC50s, as revealed by ABTS assays, were 9.0, 96.2, 23.9, and >1000, respectively [29,33,47,58], suggesting that the antioxidant activity of *P. edulis* is more than equal to that of other *Passiflora* species.

The polyphenols in *P. edulis* seeds contain a large amount of piceatannol, which has been reported to have antioxidant activity [46,59]. It is, therefore, considered to be responsible for the antioxidant activity of the seed extracts. In a rat model subjected to streptozotocin-induced oxidative stress, ingestion of ethanol extracts from *P. edulis* peel and seeds had a protective effect on the heart, liver, and kidneys against oxidative stress by enhancing superoxide dismutase levels and decreasing 2-thiobarbituric acid reactive substance levels [60]. Furthermore, attempts have been made to microencapsulate *P. edulis* peel and seed extracts in order to maintain and enhance their antioxidant activity in vivo [48]. The microencapsulated extracts showed that their antioxidant activity remained at 60% of the pre-digestion level after the digestion process [48]. Another study demonstrated that encapsulation of *P. edulis* seed extract using acylated rice starch also maintained antioxidant activity [61]. Hence, microcapsule technology may represent an effective means to transport the extracts into the body while maintaining their activity or to transport them to specific locations in the body to exert their functions.

The high antioxidant activity of *P. edulis* seed extract has also been applied to processed foods. The addition of an ethanol extract of *P. edulis* seeds has been studied to prevent lipid oxidation in dairy beverages containing sesame seed oil, which is rich in omega-3 fatty acids; the addition of the extract increased the oxidative stability of the lipids [62]. Oil extracted from *P. edulis* contains polyphenols as well as α- and β-tocopherol, and extracted oil containing these compounds has been reported to have high antioxidant activity [37]. In addition, oil extracted from *P. edulis* seeds showed the highest antioxidant activity among the extracted oils of plant seeds such as *Caryocar brasiliense* Camb., *Orbignya phalerata* Mart. and *Mauritia flexuosa* L., which grow in the Amazon [63]. These observations suggest that *P. edulis* seed oil can also be used as an antioxidant agent.

### 3.2. Effect on Skin

In vitro experiments have shown that ethanol extract of *P. edulis* seeds increases collagen production when applied to dermal fibroblasts [39]. In addition, ethanol extracts of *P. edulis* seeds exhibit inhibitory activity against collagenase and elastase [58,64,65]. The skin that covers the surface of human bodies is composed of the epidermis, dermis, and subcutaneous tissue. Approximately 70% of the dermis is composed of collagen, and collagen and elastin play important roles in the formation of the dermis structure; however, the amounts of collagen and elastin decrease with age [66,67]. *P. edulis* seed extract increases collagen production and inhibits collagen and elastin degradation, which may contribute to the maintenance of the structure of the dermis to retain skin moisture and elasticity. The collagen synthesis-promoting effect of the seed extract disappeared when the polyphenol component in the extract was removed [39], suggesting that the polyphenol component contributes to promoting collagen production.

Skin is directly exposed to UV radiation, and solar UV radiation accelerates skin aging (photoaging), causing symptoms such as coarse wrinkling, blotchy dyspigmentation, and a rough skin texture [68]. UV irradiation increases the expression of matrix metalloproteinase-1 (MMP-1), a collagen-degrading enzyme, and promotes collagen degradation, which contributes to skin aging. *P. edulis* seeds are rich in piceatannol, which has been shown to suppress UV-induced MMP-1 expression in fibroblasts; it has been suggested that inhibition of the Janus kinase 1 (JAK1) signaling pathway by piceatannol contributes to the suppression of MMP-1 expression [69]. In keratinocytes, UV irradiation does not produce MMP-1 in keratinocytes; however, reactive oxygen species (ROS) are generated by UV irradiation. Excess ROSs in keratinocytes cause oxidative damage, decrease the levels of non-enzymatic antioxidants such as glutathione (GSH), and activate complex signaling pathways that affect fibroblasts and strongly induce MMPs [70,71]. Ethanol extract of *P. edulis* seeds or piceatannol increases GSH levels in a dose-dependent manner in keratinocytes [72]. Furthermore, MMP-1 activity increased when the medium of UV-irradiated keratinocytes was applied to fibroblasts; however, the increase in MMP-1 was suppressed in the medium of keratinocytes treated with piceatannol [72]. *P. edulis* seed extract and its polyphenolic components are expected to suppress excessive ROS increase in human skin and inhibit photoaging.

Human study has been conducted to examine the effects of *P. edulis* seed extract on skin moisture and elasticity. A randomized, placebo-controlled, double-blind study was conducted to evaluate the effects of *P. edulis* seed extract (rich in piceatannol) on the skin of healthy women [73]. The results showed that the water content and elastic recovery from the pretrial were significantly increased 8 weeks after ingestion of the seed extract compared with the placebo. These results indicate that intake of *P. edulis* seed extract containing piceatannol is effective for improving skin hydration and elasticity.

The ethanol extract of *P. edulis* seeds has also been reported to inhibit tyrosinase activity [58] and inhibit melanin synthesis when applied to melanoma cells [39]. The inhibitory effect of melanin synthesis disappeared when the polyphenol fraction in the seed extract was removed, suggesting the involvement of polyphenols such as piceatannol, which has been reported to exhibit higher tyrosinase inhibitory activity than kojic acid or resveratrol [74]. Piceatannol and other stilbene compounds have been identified not only in ethanol and acetone extracts of *P. edulis* seed, but also in extracted seed oil [44], and *P. edulis* seed oil extracted by ultrasound showed tyrosinase inhibitory activity [75]. Furthermore, nanostructured lipid carrier-based hydrogels with *P. edulis* seed oil showed high tyrosinase inhibitory activity and low skin irritation; therefore, *P. edulis* seed oil has been considered for use as a cosmetic [76]. In human studies, most subjects noticed significant improvement in acne vulgaris after 8 weeks of application of a 10% *P. edulis* seed extract cream [77]. *P. edulis* seed extract exhibits antibacterial activity against *Propionibacterium acnes* [78], and this antibacterial activity of the extract may have contributed to the improvement of acne vulgaris in the human studies. In addition, another human study demonstrated that application of a cream containing 6% *P. edulis* seed extract improved the symptoms of striae distensae, a common form of skin scarring [79]. Evidently, *P. edulis* seed extract can contribute to the improvement of skin disorders such as acne vulgaris and striae distensae when applied to the skin.

### 3.3. Effect on Fat Metabolism

The effect of *P. edulis* seed extract on fat metabolism has also been examined. In vivo experiments showed that rats fed a high-fat diet showed signs of cardiovascular disease with abnormal serum profiles, whereas high-fat diets containing ethanol extracts of *P. edulis* seed improved liver enlargement, blood triglyceride, cholesterol levels, and cardiac function [80]. It has also been demonstrated that ovariectomized mice fed a high-fat diet showed marked weight gain and visceral fat accumulation, however, these effects were significantly suppressed when mice were fed a high-fat diet containing 0.05% piceatannol extracted from *P. edulis* seeds [81]. Piceatannol is considered to be a major polyphenol that exhibits anti-obesity effects among the compounds in *P. edulis* seed extract, and compared to high-fat fed mice, intake of piceatannol-containing high-fat diets decreased the weights of liver, spleen, perigonadal, and retroperitoneal fat [82].

**Figure 1:**
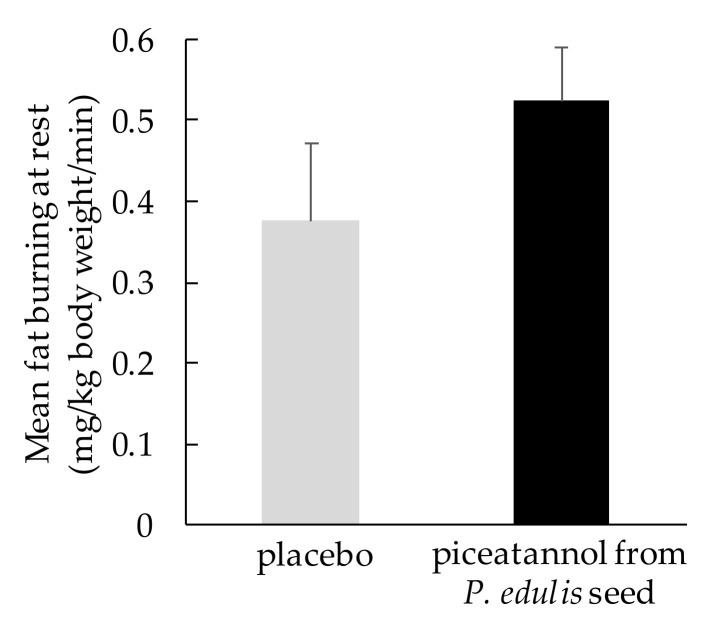
Fat-burning effect of ingestion of piceatannol from *Passiflora edulis* seed [83]. Data are shown as the mean ± SEM (*n* = 9).

Human studies on fat metabolism have been conducted, and a double-blind, placebo-controlled, crossover study showed that a food containing 10 mg piceatannol from *P. edulis* seeds for 1 week significantly reduced the respiratory quotient at rest and during very light exercise [83]. Moreover, the mean amount of fat burning at rest was increased by 39.5% during piceatannol intake compared to placebo intake (Figure 1). Another study showed that even during moderate-intensity exercise, intake of 10 mg of piceatannol from *P. edulis* seeds for 2 weeks significantly increased fat burning and decreased the respiratory quotient compared to the placebo [84]. These results show that *P. edulis* seed extract containing piceatannol can promote fat burning both at rest and during exercise.

The mechanism of action of piceatannol (which is abundant in *P. edulis* seeds) on fat metabolism has been investigated in vitro and in vivo, and enhancement of fat metabolism via SIRT and the peroxisome proliferator-activated receptor alpha (PPARα) is suggested to be the mechanism of action. The SIRT1-inducing effect of *P. edulis* seed extract and piceatannol has been reported in vitro and in vivo [41,85], and SIRT1 is thought to activate fatty acid β-oxidation by deacetylating peroxisome proliferator-activated receptor transcriptional coactivator γ1α [86]. Furthermore, piceatannol has been reported to increase PPARα in fatty liver-induced HepG2 hepatocytes [87]. Treatment of HepG2 cells with piceatannol increased PPARα, farnesoid X receptor, and carnitine palmitoyltransferase 1α and promoted β-oxidation of fatty acids. In in vivo experiments, male C57BL/6J mice fed piceatannol orally for 4 weeks showed an increase in PPARα as well as induction of SIRT1 expression [85]. In addition, piceatannol has been shown to strongly inhibit lipid synthesis and fat accumulation in human mesenchymal stem cells by suppressing the expression of fatty acid synthase and glucose transporter type 4, which are important factors in the adipogenic pathway [88]. Piceatannol improves fat metabolism through various pathways related to fat metabolism, and *P. edulis* seed extract containing piceatannol may improve fat metabolism in a similar manner.

It has been reported that not only the polyphenols in *P. edulis* seeds, but also those in extracted oil, are expected to have an inhibitory effect on fat accumulation. *P. edulis* seed oil extracted with hexane contains high amounts of linoleic and oleic acids, and the administration of this oil resulted in significant reductions in triglycerides, total cholesterol, and low-density lipoprotein-cholesterol in high-fat-diet-induced rats [35], suggesting that *P. edulis* oil is also expected to have anti-obesity effects.

### 3.4. Hypoglycemic Effect

Anti-diabetic effects of *P. edulis* seeds have been investigated, and oral administration of *P. edulis* peel and seed extract for >7 days was reported to significantly improve blood glucose levels in a rat model subjected to streptozotocin-induced oxidative stress [60]. Experiments using a genetic diabetic mouse model (*db/db* mice) also showed a significant reduction in blood glucose levels after a single dose of both *P. edulis* seed extract and its abundant component, piceatannol [40]. Regarding the mechanism of blood glucose regulation by *P. edulis* seed extract, a study examined the blood glucose-lowering effect of piceatannol from *P. edulis* seeds in freely moving healthy rats [89]. In this study, intravascularly administered piceatannol reduced blood glucose levels during both fasting and glucose tolerance tests, and piceatannol increased the insulin secretion index during the glucose tolerance test, suggesting that piceatannol from *P. edulis* seed improves glucose tolerance by promoting the initial secretion of insulin. In C57BL/6J mice fed a high-fat diet, administration of 10 mg piceatannol/kg body weight/day for 4 weeks decreased the area under the curve of blood glucose during the oral glucose tolerance test [85]. In this study, piceatannol increased the levels of insulin receptors and AMP-activated protein kinase in the liver and increased the levels of Sirt1, Sirt3, Sirt6, and two downstream targets of SIRTs, peroxisome proliferator-activated receptor gamma coactivator 1-alpha, and forkhead box O1. Evidently, piceatannol-rich seed extract can improve blood glucose levels via factors related to SIRTs and its downstream targets, as well as insulin signaling.

The seed extracts of *P. pinnatistipula* and *P. tripartita* have also exhibited α-amylase and α-glucosidase inhibitory activity in vitro [33]; however, no reports have verified its hypoglycemic effect in vivo or in human studies. Meanwhile, among the polyphenols detected in *P. edulis* seeds, stilbenes—such as piceatannol—have been shown to exhibit α-glucosidase inhibitory activity [43]. Although it remains unclear whether *P. edulis* seed has a stronger hypoglycemic effect than other species, *P. edulis* seed has shown many positive results and can be expected to exhibit hypoglycemic effects.

The effect of seed extract on glucose metabolism has been studied in human trials. Intake of 20 mg/day of piceatannol from *P. edulis* seed for 8 weeks in overweight men reduced serum insulin levels, homeostasis model assessment-insulin resistance, blood pressure, and heart rate [90]. *P. edulis* seeds are expected to be effective in improving insulin sensitivity.

### 3.5. Other Physiological Effects

The antihypertensive potential of *P. edulis* seeds was evaluated in vivo [49]. This investigation demonstrated that the ethanolic extracts obtained from *P. edulis* f. *edulis* seeds prevented hypertension induced by nitric oxide deficiency in rats. The mechanism of the antihypertensive effect of *P. edulis* seed extracts was suggested to be the synthesis of nitric oxide and inhibition or antagonism of angiotensin-II.

The anti-cancer potential of *P. edulis* seeds has also been investigated. It has been reported that *P. edulis* seed extract inhibits cancer cell proliferation via human glyoxalase I, the rate-limiting enzyme for the detoxification of methylglyoxal in both NCI-H522 cells and HCT116 cells [45]. *P. edulis* seed extract by ethanol also shows antitumor activity in MCF-7 cells, and the mechanism of antitumor activity is suggested to be induction of apoptosis via the mitochondrial pathway [91]. An in vivo study demonstrated that when an aqueous extract of *P. edulis* seeds was administered for 10 weeks, the extract affected the protein levels of p21, cyclin D1, and cyclin-dependent kinase 4; delayed disease progression in the transgenic adenocarcinoma of the mouse prostate model; and decreased the incidence of preneoplastic lesions [92]. A number of preclinical studies have shown that piceatannol can prevent the growth of cancers in various organs [93]. Cumulatively, this evidence shows that *P. edulis* seed extracts are a potential source of anti-cancer activity.

## 4. Safety of *P. edulis* Seed Extract

Passion fruit seeds are eaten raw with pulp and have been consumed for a long time. There have been many animal studies in which the seed extracts have been ingested, and several human studies have been conducted using seed extracts; however, there have been no reports of serious side effects or adverse events. In a human study, 11 healthy adults consumed a beverage containing 27.3 g of *P. edulis* seed extract (containing 100 mg of piceatannol) daily for 4 weeks to verify the safety of consuming *P. edulis* seed extract [94]. In this study, no subjects had abnormal changes in physical examinations, hematological analysis, blood biochemical tests, or urine analysis, and there were no adverse events reported during the study. Another study involved 11 healthy adults who consumed a beverage containing 8.4 g of *P. edulis* seed extract (containing 30 mg of piceatannol) for 90 d. No abnormal changes were observed in physical examinations, blood biochemical tests, or urine analysis, indicating the safety of *P. edulis* seed extract even with long-term intake [95]. The safety of the application of creams containing *P. edulis* seed extract has been examined, and an 8-h application of the extract cream with a patch cover on the lower arm of each participant showed no signs or symptoms of irritation [77]. Although further experiments on the safety of *P. edulis* seed extracts are desirable as the components in the extracts can vary depending on the extraction method, current reports have shown that ingestion and application of *P. edulis* seed extracts have been safe and can be expected to be used for food and cosmetic applications.

## 5. Conclusions

Passion fruit is a popular fruit that is consumed worldwide. *P. edulis* is widely used in processed foods such as juice, and the seeds are often discarded as a by-product. However, the industrial use of seeds is desired from the viewpoint of waste reduction, and various studies have been conducted on the industrial use of passion fruit seeds. *P. edulis* seeds contain many types of nutrients, and the lipids are rich in polyunsaturated fatty acids such as linoleic acid. In addition, the seeds contain many kinds of polyphenols and especially stilbenes, including piceatannol which is a characteristic compound of *P. edulis* seeds with a higher content than in other edible plants.

Various studies have been conducted on the physiological functions of *P. edulis* seeds, which are rich in polyphenols and exhibit strong antioxidant activity. For the skin, *P. edulis* seed extracts have skin-protective effects, such as promoting collagen synthesis and increasing the level of intracellular antioxidants, and human studies have shown that intake of seed extract improves skin moisture and elasticity. In addition to its applications in food, the seed extract has been shown to reduce acne vulgaris when applied to the skin and is expected to be used in cosmetics. Seed extract is also expected to improve fat metabolism, and the fat-burning effects of *P. edulis* seed extract have been demonstrated in human trials. The hypoglycemic effect and other physiological functions have been shown in in vivo studies, and verification of the effect in human studies is a topic for future research.

*P. edulis* seeds are beginning to be used in foods and cosmetics; however, to expand the industrial use of *P. edulis* seeds further research is required to elucidate the physiological functions of *P. edulis* seeds. The accumulation of evidence demonstrating the beneficial functions of *P. edulis* seeds in human studies will serve to increase their attractiveness as an ingredient, and thus increase the chances of their use in various processed foods and cosmetics. In addition, it will be important to develop processing technologies to extract the functional components more efficiently from *P. edulis* seeds and with higher yields. Thus, further investigation of the physiological functions and mechanisms of the components in *P. edulis* seeds. The development of processing methods for *P. edulis* seed is expected to expand the industrial applications of *P. edulis* seeds.

## Figures and Tables

**Table 1:** Nutrient analysis of *Passiflora edulis* seeds.

Components	*P. edulis*	*P. edulis*	*P. edulis* f. *flavicarpa*	*P. edulis* f. *edulis*
Nutrient Content (g/100 g Seeds on Dry Basis)				
Protein	13.99 ± 0.11	17.57 ± 0.31	13.07 ± 0.12	13.23 ± 0.48
Lipids	32.65 ± 0.45	31.16 ± 0.31	12.31 ± 0.68	14.94 ± 0.41
Carbohydrates	51.87 ± 0.00	49.44 ± 1.16	71.07 ± 0.00	69.98 ± 0.00
Ash	1.49 ± 0.01	1.82 ± 0.04	3.56 ± 0.05	1.85 ± 0.06
Dietary fiber	48.18 ± 0.64	na	65.60 ± 0.52	55.06 ± 0.35
Mineral Content (mg/100 g Seeds on Dry Basis)				
Sodium	241.7 ± 83.3	na	3.46 ± 0.07	4.80 ± 0.03
Magnesium	138.3 ± 220.5	na	150 ± 1.10	290 ± 1.80
Potassium	352.5 ± 144.3	na	760 ± 6.40	112 ± 3.00
Calcium	173.1 ± 294.8	27.46 ± 2.66	30.00 ± 0.35	6.00 ± 0.02
Phosphorus	115.3 ± 178.8	240.05 ± 7.78	310 ± 2.05	63.00 ± 1.19
Iron	6.2 ± 11.7	7.27 ± 0.27	5.20 ± 0.02	4.30 ± 0.03
Copper	1.4 ± 10	0.89 ± 0.04	0.90 ± 0.02	0.70 ± 0.02
Manganese	na	1.16 ± 0.02	2.20 ± 0.05	2.30 ± 0.03
Zinc	5.6 ± 24.7	3.72 ± 0.13	4.10 ± 0.09	4.60 ± 0.05
Reference	[26]	[29]	[30]	[30]

na, not analyzed.

**Table 2:** Lipid compositions of oil extracted from *Passiflora edulis* seeds.

Components	*P. edulis* f. *flavicarpa*	*P. edulis*	*P. edulis*	*P. edulis*
Fatty acid profile (%)				
Myristic	trace	0.10 ± 0.00	0.08	0.1
Palmitic	9.73 ± 0.01	11.00 ± 0.17	10.77	11.72
Palmitoleic	0.11 ± 0.01	0.22 ± 0.01	0.18	0.34
Stearic	2.58 ± 0.01	3.29 ± 0.31	2.98	2.84
Oleic	13.83 ± 0.04	16.84 ± 0.36	16.06	14.31
Vaccenic	na	0.17 ± 0.00	na	na
Linoleic	73.14 ± 0.05	67.39 ± 0.54	69.22	68.39
α-Linolenic	0.41 ± 0.00	0.56 ± 0.03	0.17	0.54
Araquidic	0.10 ± 0.01	na	0.43	0.16
Gadoleic	na	na	na	0.15
Eicosenoic	0.10 ± 0.00	na	na	na
Behenic	na	0.10 ± 0.00	na	0.24
Cetoleic	na	na	na	1.15
Total SFA (%)	12.41	14.69 ± 0.12	14.36	15.06
Total MUFA (%)	14.04	17.18 ± 0.47	16.24	15.95
Total PUFA (%)	73.55	68.12 ± 0.58	69.39	68.93
Reference	[10]	[29]	[34]	[35]

na, not analyzed; SFA, saturated fatty acid; MUFA, monounsaturated fatty acid; PUFA, polyunsaturated fatty acid.

**Table 3:** Polyphenols detected in *Passiflora edulis* seeds.

Compound	Species	Extraction Solvent	Reference
**Stilbene**			
Piceatannol	*P. edulis* (purple)	80% ethanol	[40]
	*P. edulis*	80% ethanol	[41]
	*P. edulis* (yellow)	ethanol	[29]
	*P. edulis*	79% ethanol	[42]
	*P. edulis*	95% ethanol	[43]
	*P. edulis*	ethanol and acetone	[44]
	*P. edulis*	35% ethanol	[45]
	*P. edulis*	90% ethanol	[46]
	*P. edulis*	70% acetone	[39]
Resveratrol	*P. edulis*	ethanol and acetone	[44]
	*P. edulis*	35% ethanol	[45]
	*P. edulis*	90% ethanol	[46]
	*P. edulis*	70% acetone	[39]
Scirpusin B	*P. edulis*	95% ethanol	[43]
	*P. edulis*	35% ethanol	[45]
	*P. edulis*	80% ethanol	[41]
	*P. edulis*	90% ethanol	[46]
Isorhapontigenin	*P. edulis*	35% ethanol	[45]
Rhapontigenin	*P. edulis*	35% ethanol	[45]
Cassigarol D	*P. edulis*	95% ethanol	[43]
Cyperusphenol B	*P. edulis*	95% ethanol	[43]
Cyperusphenol D	*P. edulis*	95% ethanol	[43]
Astringin	*P. edulis*	35% ethanol	[45]
Piceid	*P. edulis*	35% ethanol	[45]
Pinostilbene	*P. edulis*	35% ethanol	[45]
Gnetin C	*P. edulis*	35% ethanol	[45]
**Phenolic acid**			
Caffeic acid	*P. edulis*	35% ethanol	[45]
	*P. edulis* (purple)	40% methanol	[47]
Chlorogenic acid	*P. edulis* (purple)	40% methanol	[47]
Ferulic acid	*P. edulis* (purple)	40% methanol	[47]
Gallic acid	*P. edulis* (purple)	40% methanol	[47]
Rosmarinic acid	*P. edulis* (purple)	40% methanol	[47]
Coumarin	*P. edulis*	50% ethanol, 70% acetone	[38]
p-coumaric acid	*P. edulis* f. *edulis*	ethanol	[48]
	*P. edulis*	35% ethanol	[45]
**Flavonoid**			
Epicatechin	*P. edulis*	35% ethanol	[45]
Quercetin	*P. edulis*	40% methanol	[47]
	*P. edulis* f. *edulis*	ethanol	[48]
Rutin	*P. edulis* f. *edulis*	ethanol	[48]
Isoquercetin	*P. edulis* f. *edulis*	ethanol	[48]
Kaempferol	*P. edulis* f. *flavicarpa*	ethanol	[30]
Malvidin 3,5-diglucoside	*P. edulis* f. *flavicarpa*, *P. edulis* f. *edulis*	ethanol	[30]
Orientin	*P. edulis* f. *edulis*	97% ethanol	[49]
Isoorientin	*P. edulis* f. *edulis*	97% ethanol	[49]
Vitexin	*P. edulis* f. *edulis*	97% ethanol	[49]
Isovitexin	*P. edulis* f. *edulis*	97% ethanol	[49]
